# Mechanistic Insights into Oxygen Release from Layered Oxide Cathodes for Reliable Sodium‐Ion Batteries

**DOI:** 10.1002/smll.74070

**Published:** 2026-06-08

**Authors:** Toshiya Takuwa, Masataka Yoshimoto, Yuta Kimura, Koji Amezawa, Hitoshi Abe, Yasuhiro Niwa, Satoshi Hiroi, Koji Ohara, Huu Duc Luong, Yasunobu Ando, Yoshitaka Tateyama, Takashi Nakamura

**Affiliations:** ^1^ Graduate School of Engineering Nagoya University Nagoya Japan; ^2^ Institute of Materials and Systems for Sustainability Nagoya University Nagoya Japan; ^3^ Institute of Multidisciplinary Research For Advanced Materials Tohoku University Sendai Japan; ^4^ Institute of Materials Structure Science High Energy Accelerator Research Organization Tsukuba Ibaraki Japan; ^5^ Materials Structure Science Program The Graduate University For Advanced Studies (SOKENDAI) Tsukuba Ibaraki Japan; ^6^ Graduate School of Science and Engineering Ibaraki University Mito Ibaraki Japan; ^7^ Faculty of Materials for Energy Shimane University Matsue Shimane Japan; ^8^ Laboratory For Chemistry and Life Science Institute of Science Tokyo Yokohama Kanagawa Japan

**Keywords:** cathode active materials, oxygen release, sodium‐ion batteries

## Abstract

Oxygen release from cathode materials is a serious atomistic‐scale problem causing thermal runaway of rechargeable batteries. Understanding the mechanism of oxygen release and establishing design principles for robust cathode materials are crucial for the practical implementation of sodium‐ion batteries (SIBs). Herein, it is successful, for the first time, to the elucidate mechanism of oxygen release from SIB cathode Na_2/3_Ni_1/3_Mn_2/3_O_2_ by combining experimental and theoretical studies and to provide evidences for the superior stability of SIBs. Coulometric titration, X‐ray absorption spectroscopy and defect‐chemical/thermodynamic analyses reveal that the oxygen release energy of Na_2/3_Ni_1/3_Mn_2/3_O_2_ is approximately 1.7 eV which is higher than those of high‐Ni lithium‐ion battery cathodes. Density functional theory calculations strongly suggest that high‐valent Ni formed by Na‐deficiency destabilizes lattice oxygen in Na_2/3_Ni_1/3_Mn_2/3_O_2_ and facilitates oxygen release. The combined experimental and theoretical study presented here enables a deeper mechanistic understanding of oxygen release from cathode materials and offers valuable design insights for next‐generation batteries.

## Graphical abstract

Oxygen release behavior of Na_2/3_Ni_1/3_Mn_2/3_O_2_ is investigated, and mechanistic insight into oxygen release from cathode materials are discussed by integrating experimental investigations with theoretical calculations. Comparison of lattice oxygen stability of Na_2/3_Ni_1/3_Mn_2/3_O_2_ and that of Li‐ion battery cathodes are summarized.

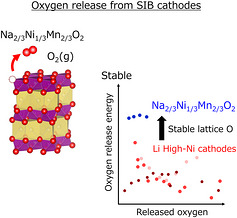

## 1 Introduction

Transition metal oxides are widely accepted as electrode materials for next‐generation batteries such as all‐solid‐state, Na‐ion, Mg‐ion, and Fluoride‐ion batteries [1, 2, 3, 4, 5, 6, 7]. Although oxide‐based cathode active materials achieve excellent electrochemical properties, they inevitably lose lattice oxygen because of inherent redox activity of transition metals. Such oxygen release, oxygen vacancy formation, degrades electrochemical performance [8, 9, 10, 11], and even worth, cause catastrophic thermal runaway by exothermic reaction with organic liquid electrolytes [12, 13, 14]. Safety concerns of sodium‐ion batteries (SIBs) are widely investigated for practical applications [15, 16, 17, 18]. Thermal runaway behavior of an SIB composed of layered oxide cathodes and lithium‐ion batteries (LIBs) composed of LiNi_0.5_Co_0.2_Mn_0.3_O_2_ (NCM523) and LiFePO_4_ (LFP) cathodes were investigated in an explosion‐proof chamber, and it was found that the risk of thermal runaway is LIB (NCM523) > SIB (layered oxide cathode) > LIB (LFP) [15]. This strongly suggests that the safety of a whole battery system essentially depends on the stability of cathode active materials. Moreover, even if flammable liquid electrolytes are replaced by solid electrolytes, electrode performance degradation and expansion of a battery package due to gas generation from cathode materials remain serious problems [19, 20, 21]. Therefore, understanding detailed mechanism of lattice oxygen release from oxide‐based cathode materials is of importance for robust and safe next‐generation batteries.

So far, oxygen release was investigated by both experiments and theoretical calculations. Trade‐off relation between thermal stability and discharge capacity of practical Li(Ni,Co,Mn)O_2_ cathodes were systematically investigated [22]. In‐situ and operando measurement techniques are powerful tool to investigate oxygen release and phase change of charged cathode materials [23, 24, 25, 26, 27]. For instance, K. W. Nam et al., reported oxygen release by thermal decomposition of layered rock‐salt oxides to spinel and disordered rock‐salt oxides by combining in‐situ XRD, mass spectroscopy, XAS and transmission electron microscopy [27]. Most of experimental works focus on oxygen release/gas generation by thermal decomposition of charged cathode materials by external heating [27, 28, 29]. In contrast, theoretical calculation quantitatively report oxygen vacancy formation energy of oxide based Li‐cathode and Na‐cathodes [30, 31, 32, 33, 34, 35, 36, 37, 38, 39, 40, 41, 42]. Oxygen vacancy formation energy is important clue to investigate oxygen release mechanism, however, the reported energies scattered in a wide range because of differences in calculation method and assumptions (summarized in our previous study [43]). Moreover, the situations assumed in experimental studies, thermal decomposition, and that in theoretical calculations, lattice oxygen loss while retaining the original structure, are completely different. Such critical inconsistency between experiments and calculations hinders a deep understanding of lattice oxygen stability in cathode materials. To link experimental works and theoretical calculations, the authors developed methodology to investigate oxygen release behavior of oxide cathode materials by applying oxygen coulometric titration and thermodynamic analysis [43, 44, 45, 46]. It was revealed that high‐Ni NCM cathodes easily lose lattice oxygen by reduction of high‐valent Ni, and necessary energy for oxygen release is determined by reduction species. Oxygen release by Ni reduction requires 0.5–1.4 eV, while oxygen release by Co reduction requires more than 2.4 eV [43].

In this work, as a first demonstration of lattice oxygen stability in sodium‐ion battery cathodes, detailed mechanism of lattice oxygen release from a solium‐ion battery cathode P2‐Na_2/3_Ni_1/3_Mn_2/3_O_2_ was investigated by the oxygen coulometric titration, X‐ray diffractometry (XRD), X‐ray absorption spectroscopy (XAS), and defect‐chemical and thermodynamic analyses. Moreover, density functional theory (DFT) calculations provide important insights into changes of electronic structures and defect formation energy. From rational combination of experiments and calculations, detailed oxygen release mechanism is discussed.

## 2 Results and Discussion

### 2.1 Characterization of Pristine Na_2/3_Ni_1/3_Mn_2/3_O_2_

**FIGURE 1:**
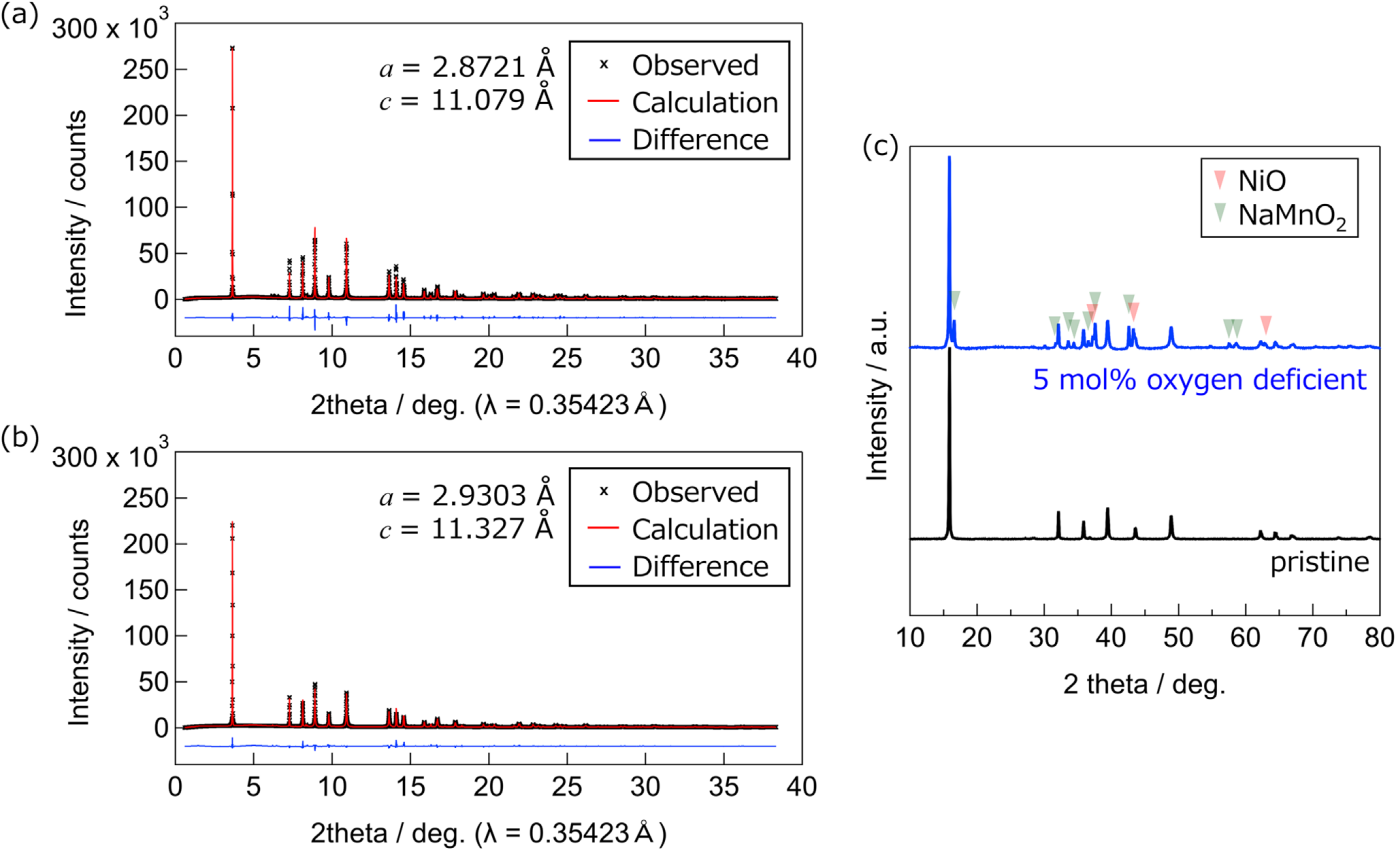
Synchrotron XRD patterns and Rietveld refinement results of (a) the pristine Na_2/3_Ni_1/3_Mn_2/3_O_2_ and (b) the 1mol% oxygen‐deficient Na_2/3_Ni_1/3_Mn_2/3_O_2_. (c) XRD patterns of the pristine and the 5 mol% oxygen‐extracted Na_2/3_Ni_1/3_Mn_2/3_O_2_ evaluated by Cu Kα radiation.

X‐ray diffraction patterns of as synthesized P2‐Na_2/3_Ni_1/3_Mn_2/3_O_2_ evaluated by synchrotron X‐ray are shown in Figure 1a. The diffraction patterns of the pristine Na_2/3_Ni_1/3_Mn_2/3_O_2_ are well explained by a space group *P63/mmc*, and the structure parameters determined by Rietveld analysis were summarized in Table S2. SEM images and EDS mapping of the pristine Na_2/3_Ni_1/3_Mn_2/3_O_2_ particles are shown in Figure S1. Uniform particles and elemental distribution were confirmed. The electrochemical performance of as prepared Na_2/3_Ni_1/3_Mn_2/3_O_2_ was briefly checked by Na metal half‐cell composed of the cathode composite electrode/1 M NaPF_6_ EC‐DEC (1:1 vol.)/Na metal. The pristine Na_2/3_Ni_1/3_Mn_2/3_O_2_ used in this work achieved reasonable charge/discharge performance as shown in Figure S2 [47, 48].

### 2.2 Oxygen Release From Na_2/3_Ni_1/3_Mn_2/3_O_2_

**FIGURE 2:**
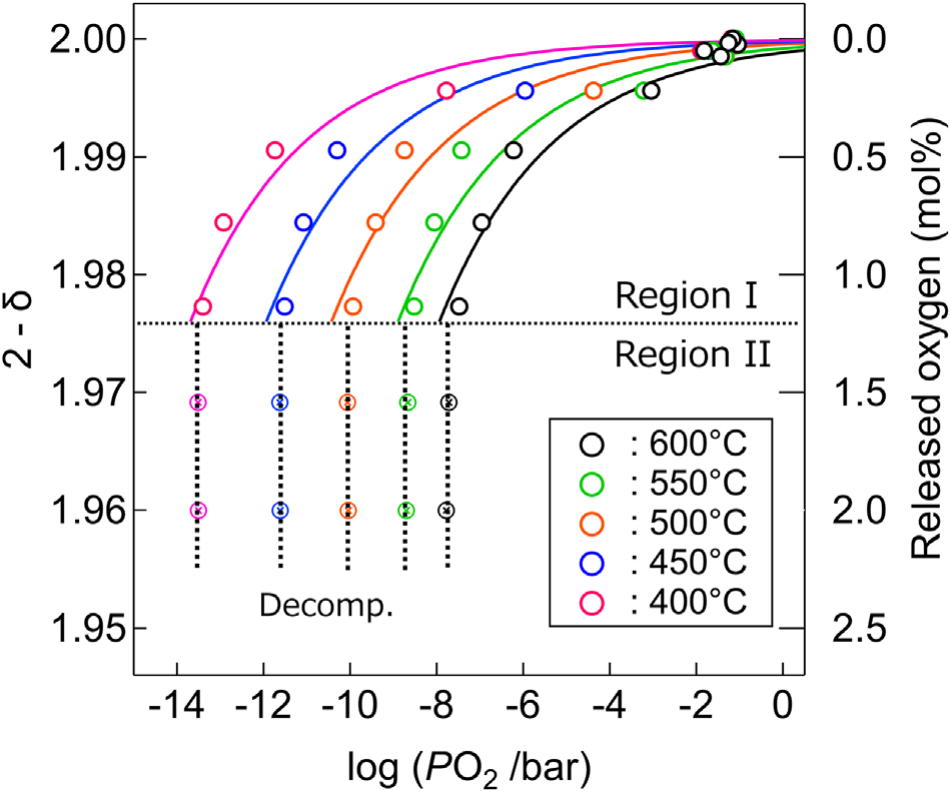
Oxygen release behavior of Na_2/3_Ni_1/3_Mn_2/3_O_2_. Region I represents solid‐solution‐like oxygen release and Region II represents reduction decomposition. Dotted lines in the region II represent *P*(O_2_) of reduction limit of Na_2/3_Ni_1/3_Mn_2/3_O_2_. Solid curves in the Region I represent calculation results of the defect equilibrium model, eq. 7, with $\Delta G_{\mathrm{ox}}^{\circ }$ in Table 1.

Figure 2 shows the oxygen release behavior of P2‐Na_2/3_Ni_1/3_Mn_2/3_O_2_ evaluated by the oxygen coulometric titration technique, assuming that the pristine Na_2/3_Ni_1/3_Mn_2/3_O_2_ is almost oxygen‐stoichiometric. This diagram represents thermodynamic relation between oxygen composition as functions of temperature and oxygen chemical potential, log *P*(O_2_). In the region I, lattice oxygen was released in a solid‐solution‐like manner without destroying the original crystal structure. In contrast, reduction decomposition proceeded in the region II, where the main phase coexists with reduction decomposition phases, and the equilibrium oxygen chemical potential was fixed in accordance with the Gibbs phase rule. The maximum concentration of oxygen vacancy in P2‐Na_2/3_Ni_1/3_Mn_2/3_O_2_ is considered to be approximately 1.2 mol%, and further oxygen release causes reduction decomposition. NaNiO_2_ and NiO were clearly detected from XRD pattern of 5 mol% oxygen‐extracted Na_2/3_Ni_1/3_Mn_2/3_O_2_ as shown in Figure 1c. 1 mol% oxygen‐deficient Na_2/3_Ni_1/3_Mn_2/3_O_2_ which is within the region I showed no XRD peak of impurity phases and slightly larger lattice parameters due to oxygen release was confirmed (Figure 1b and Table S2). These strongly support the above discussion that solid‐solution‐like oxygen release proceeded in the Region I. Based on the above discussion, lattice oxygen release from Na_2/3_Ni_1/3_Mn_2/3_O_2_ can be categorized into two regions as follows:

Region I: Oxygen vacancy formation (solid‐solution reaction)

(1) $$Na_{2/3}Ni_{1/3}Mn_{2/3}O_{2}\to Na_{2/3}Ni_{1/3}Mn_{2/3}O_{2-\delta }+\frac{\delta }{2}O_{2}\left(g\right)$$

Region II: Reduction decomposition (multi‐phase reaction)

(2) $$Na_{2/3}Ni_{1/3}Mn_{2/3}O_{2}\to \frac{2}{3}\mathrm{NaMn}O_{2}+\frac{1}{3}\mathrm{NiO}+\frac{1}{6}O_{2}\left(g\right)$$

### 2.3 Charge Compensation During Oxygen Release

**FIGURE 3:**
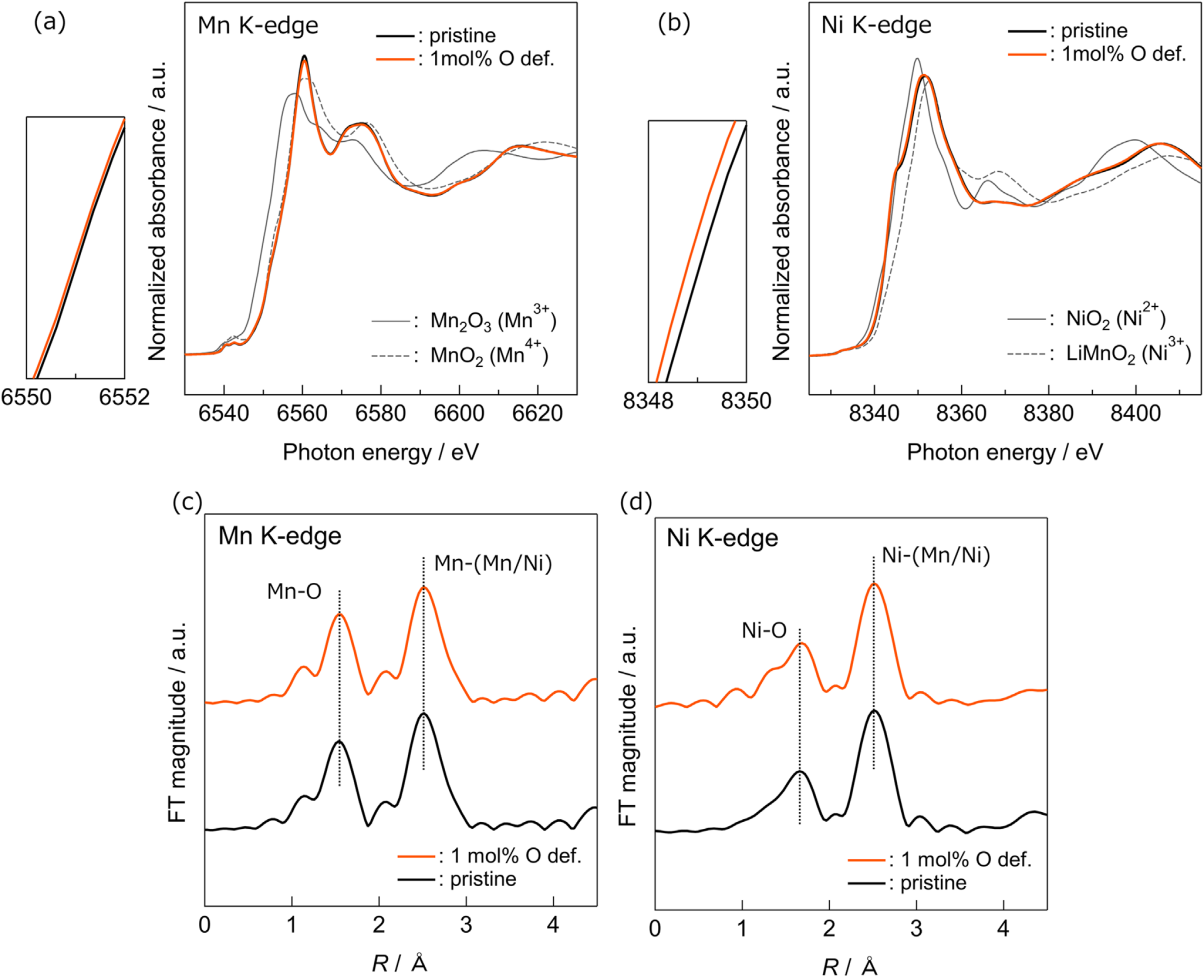
X‐ray absorption spectra of the pristine and the 1 mol% oxygen‐deficient Na_2/3_Ni_1/3_Mn_2/3_O_2_ at (a) Mn K‐edge and (b) Ni K‐edge. Radial distribution function of the pristine and the oxygen‐deficient samples obtained by EXAFS spectra at (c) Mn K‐edge and (d) Ni K‐edge.

To investigate reduction elements during oxygen release, X‐ray absorption spectroscopy was carried out on the pristine and the 1mol% oxygen‐deficient Na_2/3_Ni_1/3_Mn_2/3_O_2_. Figure 3 shows X‐ray absorption spectra at (a) Mn K‐edge and (b) Ni K‐edge. Compared with reference materials such as Mn_2_O_3_ (Mn^3+^), MnO_2_ (Mn^4+^), NiO (Ni^2+^) and LiNiO_2_ (Ni^3+^), the nominal oxidation state of Mn and Ni in the pristine Na_2/3_Ni_1/3_Mn_2/3_O_2_ are almost Mn^4+^ and Ni^2+^ which is consistent with literatures [47, 48]. These also suggest that the pristine sample is almost oxygen‐stoichiometric, although the sample was quenched in Ar atmosphere. After oxygen release, the absorption edge at Ni K‐edge clearly shifted to lower energy, and that of Mn K‐edge shifted slightly to the lower energy, suggesting that the charge balance of oxygen release was maintained mainly by Ni reduction and weak Mn reduction was also contributed. Ni L‐edge and Mn L‐edge spectra summarized in Figure S3 showed similar reduction behavior.

Figure 3c,d show the Fourier transformed Mn K‐edge and Ni K‐edge EXAFS spectra which indicate the radial distribution function of the local atomic environment around Mn and Ni. The peak at around 1.5 Å represents the distance to oxygen, Mn–O and Ni–O distances, and that of the peak at around 2.5 Å represents the distance to the neighboring transition metals, Mn–Mn/Ni and Ni–Mn/Ni distances. Oxygen deficient sample showed slightly larger Ni‐O distance, while Mn‐O, Mn‐Mn/Ni and Ni‐Mn/Ni distances seems to be almost invariant. This local structural change is qualitatively consistent with the lattice parameter changes evaluated by XRD.

Although clear Ni reduction was observed, reduction of Ni^2+^ to Ni^+^ is unrealistic in Ni‐containing compounds. One possible hypothesis for apparent Ni reduction in Na_2/3_Ni_1/3_Mn_2/3_O_2_ by oxygen release is that Na vacancy ($V_{\mathrm{Na}}^{\prime }$) was formed unexpectedly during high temperature synthesis because of high volatility of Na, and a part of Ni was oxidized to maintain charge neutrality. High‐valent Ni was selectively reduced to Ni^2+^ by oxygen release. Similarly, clear influence of negative alkaline defects, Li vacancy ($V_{\mathrm{Li}}^{\prime }$) and/or Li at transition metal site (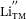), and high‐valent Ni on reduction behavior of Li_x_Ni_1/3_Co_1/3_Mn_1/3_O_2_ was confirmed in our previous study [45]. Because expected Na vacancy concentration is very small, clear evidence of Na vacancy was not obtained by structure analysis. Assuming a defect pair of Na vacancy and high‐valent Ni is more realistic than Ni^2+^ reduction, and the validity of this hypothesis, the influence of Na‐deficiency on reduction behavior, is tested by DFT calculations in 2.5. Because initial oxygen release (the Region I in Figure 2) needs reduction of Ni^3+^ to Ni^2+^, one can avoid this initial oxygen release by eliminating the formation of the defect pair, $V_{\mathrm{Na}}^{\prime }$ and Ni^3+^. This is important finding for battery material production that the precise compositional control is a key for reliable cathode materials.

**FIGURE 4:**
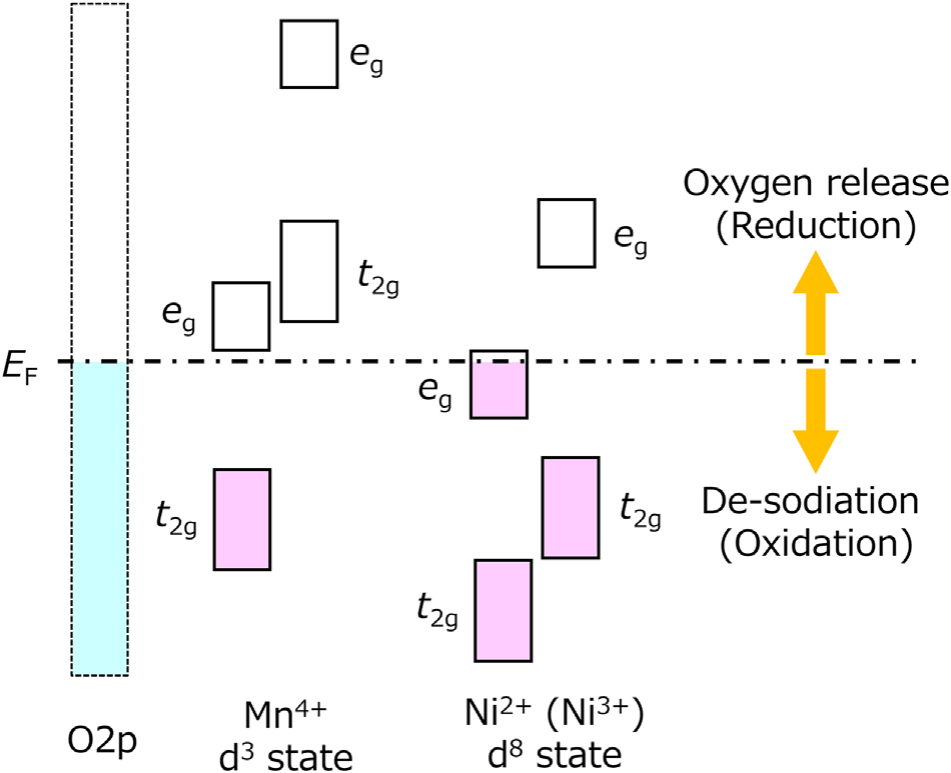
Schematic illustration of electronic structure of Na_2/3_Ni_1/3_Mn_2/3_O_2_.

Schematic electronic structures of Na_2/3_Ni_1/3_Mn_2/3_O_2_ are illustrated in Figure 4, assuming rigid‐band‐like situation. This means that the Fermi level shift upwards/downwards by reduction/oxidation due to the creation of electron/hole, while distribution of DOS remain unchanged. Although this model is just a schematic picture, this can roughly explain reduction behavior due to oxygen release and the redox behavior during charge and discharge of Na_2/3_Ni_1/3_Mn_2/3_O_2_, namely, Ni reduction during oxygen release as well as selective Ni oxidation during charge (Ni^2+^ → Ni^4+^) and selective Ni reduction during discharge (Ni^4+^ → Ni^2+^), while anion redox was also suggested at high‐voltage region [47, 48, 49].

### 2.4 Defect Chemical and Thermodynamic Analyses

For further discussion on oxygen release by oxygen vacancy formation, defect‐chemical and thermodynamic analyses were performed. Oxygen release from Na_2/3_Ni_1/3_Mn_2/3_O_2_ was investigated by assuming the equilibrium between Na_2/3_Ni_1/3_Mn_2/3_O_2_ and O_2_ molecules in the gas phase. The equilibrium reaction between the sample and O_2_(g), and its Gibbs free energy change, $\Delta G_{\mathrm{ox}}^{\circ }$, are expressed by

(3) $$O_{O}^{\times }↔V_{O}^{\cdot \cdot }+2e^{\prime }+\frac{1}{2}O_{2}\left(g\right)$$

(4) $$\Delta \;G_{\mathrm{ox}}^{\circ }=-RT\ln\left(\frac{\left[V_{O}^{\cdot \cdot }\right]{\left[e^{\prime }\right]}^{2}P{\left(O_{2}\right)}^{1/2}}{\left[O_{O}^{\times }\right]}\right)-RT\ln\frac{\gamma_{V_{O}^{\cdot \cdot }}\gamma_{e^{\prime }}^{2}}{\gamma_{O_{O}^{\times }}}$$

where defect species were expressed by the Kröger‐Vink notation and *R*, *T* and *γ* represent the gas constant, the temperature and the activity coefficient, respectively. Itinerant electron was assumed in this analysis for the simplicity, although Ni and Mn cooperatively contribute to the change neutrality (Figure 3). The charge balance and the oxygen site conservation are maintained as

(5) $$2\left[V_{O}^{\cdot \cdot }]=[e^{\prime }\right]$$

(6) $$[O_{O}^{\times }]+[V_{O}^{\cdot \cdot }]=2$$

From equations (4), (5), (6), the defect equilibrium model which represents the relation among *δ*‐*T*‐*P*O_2_ can be described by using $\Delta G_{\mathrm{ox}}^{\circ }$ as a fitting parameter.

(7) $$P\left(O_{2}\right)={\left(\frac{\left[O_{O}^{\times }\right]}{\left[V_{O}^{\cdot \cdot }\right]{\left[e^{\prime }\right]}^{2}}\exp\left(-\frac{\Delta G_{\mathrm{ox}}^{\circ }}{RT}\right)\right)}^{2}={\left(\frac{2-\delta }{4\delta^{3}}\exp\left(-\frac{\Delta G_{\mathrm{ox}}^{\circ }}{RT}\right)\right)}^{2}\;$$

here, we assume defect formation is ideal‐solution‐like, and therefore, the activity coefficients are unity. The calculated results at each temperature are shown in Figure 2 as solid lines, and $\Delta G_{\mathrm{ox}}^{\circ }$ are summarized in Table 1.The defect equilibrium model can explain oxygen release behavior in the Region I, solid‐solution‐like oxygen release, in Na_2/3_Ni_1/3_Mn_2/3_O_2_. The enthalpy change of the defect equilibrium, $\Delta H_{\mathrm{ox}}^{\circ }$, can be calculated from $\Delta G_{\mathrm{ox}}^{\circ }$ and Gibbs‐Helmholtz equation. As shown in Figure S4, linear relation between $\Delta G_{\mathrm{ox}}^{\circ }/T$ vs. 1/*T* was confirmed, and the calculated $\Delta H_{\mathrm{ox}}^{\circ }$ is approximately 1.7 eV which represents enthalpy change of oxygen vacancy formation reaction, equation 3.

**TABLE 1:** $\Delta G_{\mathrm{ox}}^{\circ }$ of Na_2/3_Ni_1/3_Mn_2/3_O_2_ at 400–600°C.

*T* [°C]	$\Delta G_{\mathrm{ox}}^{\circ }$ [eV]
600	−1.48
550	−1.47
500	−1.50
450	−1.51
400	−1.52

**FIGURE 5:**
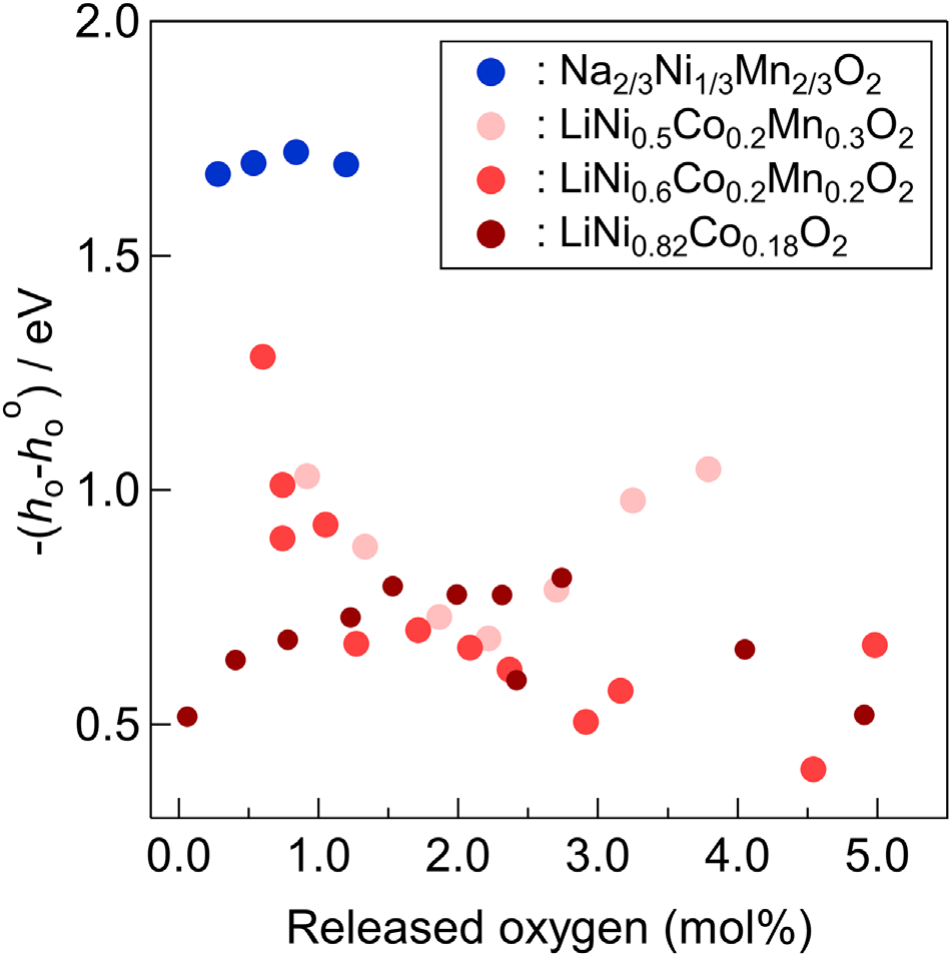
Partial molar enthalpy of oxygen as a function of the amount of released oxygen. $h_{O}-h_{O}^{\circ }$ of high‐Ni LIB cathodes were replotted from refs [43, 46].

Similarly, partial molar enthalpy of oxygen, $h_{O}-h_{O}^{\circ }$, which represents partial molar enthalpy change due to oxygen content change, was obtained from oxygen nonstoichiometric data in Figure 2 and the Gibbs‐Helmholtz equation as

(8) $$h_{O}-\;h_{O}^{\circ }=\frac{\partial }{\partial \left(1/T\right)}\;\left(\frac{R}{2}\ln P\left(O_{2}\right)\right)$$

where $h_{O}^{\circ }$ is the partial molar enthalpy at the standard state. Linear *R*/2 ln*P*(O_2_) vs. 1/*T* relation was confirmed (Figure S5), and the calculated $h_{O}-h_{O}^{\circ }$ of Na_2/3_Ni_1/3_Mn_2/3_O_2_ is approximately 1.7 eV regardless of the amount of released oxygen as shown in Figure 5. Since $\Delta H_{\mathrm{ox}}^{\circ }$ is closely related to $h_{O}-h_{O}^{\circ }$ (see the supporting information “Theoretical relation between defect equilibrium model and thermodynamic parameters”), $\Delta H_{\mathrm{ox}}^{\circ }$ obtained by defect‐ chemical analysis is almost the same with $h_{O}-h_{O}^{\circ }$ obtained by thermodynamic analysis, suggesting that oxygen vacancy formation in Na_2/3_Ni_1/3_Mn_2/3_O_2_ is ideal‐solution‐like. The most important finding of these analyses is that necessary energy of oxygen release from Na_2/3_Ni_1/3_Mn_2/3_O_2_ is larger than that of high‐Ni Li‐ion battery cathodes such as LiNi_0.5_Co_0.2_Mn_0.3_O_2_, LiNi_0.6_Co_0.2_Mn_0.2_O_2_ and LiNi_0.82_Co_0.18_O_2_ [43, 46]. This means that lattice oxygen in Na_2/3_Ni_1/3_Mn_2/3_O_2_ is more stable than that in high‐Ni cathodes. Although these evaluations are lattice oxygen stability of fully‐discharged cathodes, the present work partly supports the superior stability of SIBs to LIBs composed of high‐Ni cathodes [15]. Plausible explanation for higher stability of Na‐cathodes than high‐Ni LIB cathodes is the influence of cation disorder. In high‐Ni NCMs, Li/Ni mixing and oxygen release simultaneously proceeded because of similar ionic size of Li and Ni (Li^+^: 0.76 Å, Ni^2+^: 0.69 Å), and $h_{O}-h_{O}^{\circ }$ decrease with oxygen release. In contrast, Na/transition metal mixing does not proceed in Na layered oxides due to huge mismatch of ionic size (Na^+^: 1.02 Å and 3d transition metals: 0.5‐0.8 Å), and $h_{O}-h_{O}^{\circ }$ of Na_2/3_Ni_1/3_Mn_2/3_O_2_ shows almost constant against oxygen release.

### 2.5 Density Functional Calculations

**FIGURE 6:**
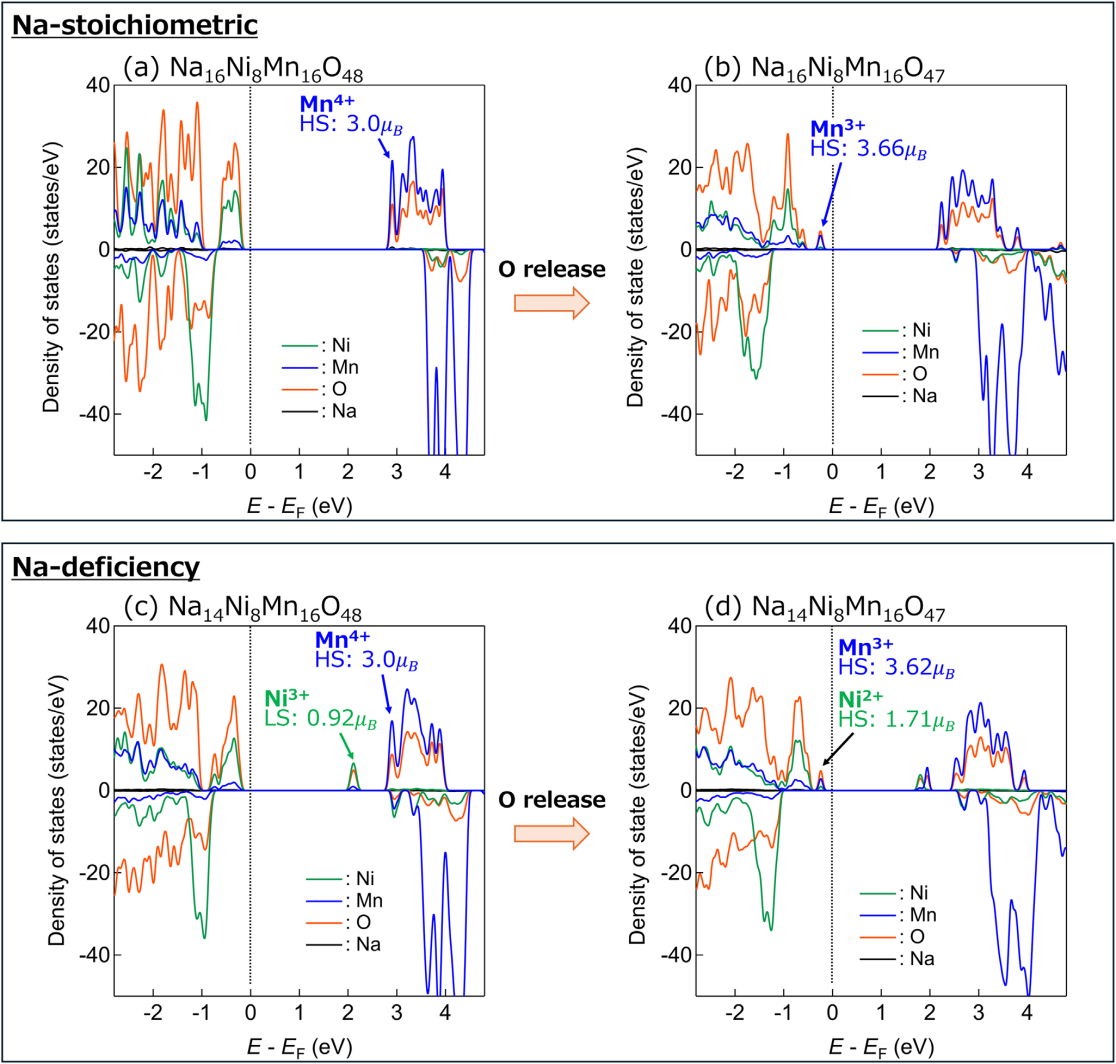
Projected density of state (PDOS) of the Na‐stoichiometric supercell (a) oxygen‐ stoichiometric Na_16_Ni_8_Mn_16_O_48_ and (b) oxygen‐extracted Na_16_Ni_8_Mn_16_O_47_. PDOS of the Na‐deficient supercell (c) oxygen‐stoichiometric Na_14_Ni_8_Mn_16_O_48_ and (d) oxygen‐extracted Na_14_Ni_8_Mn_16_O_47_.

To deepen understandings on oxygen release mechanism, DFT calculations were performed with Na‐stoichiometric supercell Na_16_Ni_8_Mn_16_O_48_ ($2\sqrt{3}\times 2\sqrt{3}\times 1$) and its oxygen‐deficient state Na_16_Ni_8_Mn_16_O_47_ at first [50, 51, 52, 53, 54]. The calculated projected densities of states (pDOS) shown in Figure 6 indicates that the former is composed of Ni^2^^+^ (d^8^, high spin, local magnetic moment of 1.7 µ_B_) and Mn^4^^+^ (d^3^, high spin, local magnetic moment of 3.0µ_B_) in principle. The conduction band minimum (CBM) mainly consists of a hybridized state of 3d Mn^4+^ and 2p O ions (Figure 6a). After oxygen release, newborn states at the valence band maximum (VBM) originate from two Mn^3+^ ions (d^4^, high‐spin, local magnetic moment: 3.66µ_B_), while Ni^2+^ states remain invariant (Figure 6b). These results imply that the charge compensation upon oxygen release from the Na‐stoichiometric Na_16_Ni_8_Mn_16_O_48_ is solely maintained by Mn reduction as

(9)

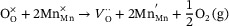

and the calculated oxygen vacancy formation energy is approximately 2.41 eV, which is much higher than the experimentally obtained oxygen release energy (1.7 eV) under thermodynamic equilibrium. The inconsistencies in the reduction element and oxygen vacancy formation energy strongly suggest that our pristine sample is not the Na‐stoichiometric Na_2/3_Ni_1/3_Mn_2/3_O_2_. As discussed in 2.4, the pristine sample tested in this work was likely to contain Na‐deficiency.

To understand the impact of Na vacancy on oxygen release behavior and lattice oxygen stability, Na‐deficient supercell Na_14_Ni_8_Mn_16_O_48_ and its oxygen‐deficient structure Na_14_Ni_8_Mn_16_O_47_ were also examined by DFT calculations. In the Na‐deficient Na_14_Ni_8_Mn_16_O_48_, CBM is mainly composed of low‐spin state Ni^3+^ (d^7^, local magnetic moment: 0.92 µ_B_) and O‐2p, while the unoccupied Mn^4+^ states are about 0.5 eV above the CBM (see Figure 6c). After an oxygen extraction, two neighboring sites of the oxygen vacancy, Ni^3+^ and Mn^4+^ ions, increases their magnetic moments by 0.7µ_B_, suggesting that Mn^4+^ (d^3^, 3.0µ_B_) and Ni^3+^(d^7^, 0.92µ_B_) are reduced to Mn^3+^ (d^4^, 3.66µ_B_) and Ni^2+^(d^8^, 1.71µ_B_), respectively. As shown in Figure 6d, VBM is mainly composed of the reduced high‐spin Mn^3+^ and O‐2p, while the reduced Ni^2+^ state locates at the deeper energy region of the valence band. This evidences that charge compensation due to oxygen release is maintained by a simultaneous reduction of Ni^3+^ and Mn^4+^ as

(10)

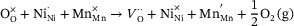




In this situation, the calculated oxygen vacancy formation energy is 1.87 eV. In the Na‐deficient situation, both reduction element and oxygen vacancy formation energy agree very well with the experimental results. This strongly suggests that unexpectedly formed negatively charged defects, such as Na vacancies, generate high‐valent Ni species, which in turn destabilize lattice oxygen. Stoichiometric Na_2/3_Ni_1/3_Mn_2/3_O_2_ is predicted to exhibit higher lattice oxygen stability than real Na_2/3_Ni_1/3_Mn_2/3_O_2_ containing trace amounts of Na defects and high‐valent Ni. These findings highlight the critical importance of strict stoichiometric control for achieving intrinsically stable cathode materials. In addition, this result demonstrates the reliability of the HSE06 method compared with the GGA+*U* method, which is strongly dependent on the choice of Hubbard term *U*, as shown in Table 2.

**TABLE 2:** Necessary energy of oxygen release of P2‐Na_2/3_Ni_1/3_Mn_2/3_O_2_.

Value [eV]	Method, condition	Refs.
1.7	Partial molar enthalpy of oxygen obtained by thermodynamic analysis on oxygen nonstoichiometry	this work
1.7	Enthalpy of oxygen vacancy formation obtained by defect‐chemical analysis on oxygen nonstoichiometry	this work
1.87	DFT calculations by HSE06 method for Na‐deficient structure, *T* = 400 K, and *P*(O_2_) = 0.2 atm	this work
2.41	DFT calculations by HSE06 method for Na‐stoichiometric Na_2/3_Ni_1/3_Mn_2/3_O_2_, *T* = 400 K, and *P*(O_2_) = 0.2atm	this work
1.93	DFT calculations by GGA+*U* (*U*_Mn_ = 3.8 eV, *U*_Ni_ = 6.1 eV) method for Na‐stoichiometric bulk	[37]
1.49	DFT calculations by GGA+*U* (*U*_Mn_ = 3.5 eV, *U*_Ni_ = 5.5 eV) method for Na‐stoichiometric surface (002)	[39]
1.29	DFT calculations by GGA+*U* (*U*_Mn_ = 3.9 eV, *U*_Ni_ = 6.2 eV) method for Na‐stoichiometric surface (002)	[42]

## 3 Conclusions

Considering both experimental results and theoretical calculations in this work, following finding are obtained. 1) Oxygen coulometric titration and defect‐chemical/thermodynamic analyses are powerful tool to understand the mechanism of oxygen release from oxide‐based cathode active materials. This methodology enables reasonable comparison of experimental results and DFT calculations which have not been performed before. 2) P2‐Na_2/3_Ni_1/3_Mn_2/3_O_2_ has higher lattice oxygen stability than high‐Ni Li‐ion battery cathodes. This supports higher safety and robustness of SIBs than high‐energy‐density LIBs. 3) Na‐stoichiometric Na_2/3_Ni_1/3_Mn_2/3_O_2_ is much more stable than Na‐deficient one. Unexpectedly introduced defect pairs like high‐valent Ni and Na vacancy significantly affect the lattice oxygen stability, because such defects may drastically change charge compensation. Control of defect species is crucially important for achieving intrinsically stable and high‐energy‐density cathode materials. Namely, robustness of cathode materials can be enhanced by careful control of alkaline composition in the synthesis process. Through this study, we successfully establishes a reliable experimental and analytical framework for the quantitative evaluation of lattice oxygen stability in oxide‐based battery materials. The combined experimental and theoretical study presented here enables a deeper mechanistic understanding of oxygen release from cathode materials and offers valuable design insights for next‐generation batteries, not only SIBs but also solid‐state batteries, magnesium‐ion batteries and halide‐ion batteries.

## Funding

This work was supported in part by JST as GteX (Grant No. JPMJGX23S4), JSPS KAKENHI (Grant Nos. 24H02203, 24H02205, 25H01979), MEXT as the “Program for Promoting Research on the Supercomputer Fugaku” (Grant No. JPMXP1020230325).

## Conflicts of Interest

The authors declare no conflicts of interest.

## Supporting information




**Supporting File**: smll74070‐sup‐0001‐SuppMat.docx.

## Data Availability

The data that support the findings of this study are available from the corresponding author upon reasonable request.
