# Supplementary material for: Mechanistic Insights into Oxygen Release from Layered Oxide Cathodes for Reliable Sodium‐Ion Batteries
Source: Small. 2026 Jun 8;22(42):e74070. doi: 10.1002/smll.74070 (PMC13410437; doi:10.1002/smll.74070)
Supplement: Supplementary file 1 — Supporting File: smll74070‐sup‐0001‐SuppMat.docx. [file SMLL-22-e74070-s001.docx]

Supporting Information

Mechanistic Insights into Oxygen Release from Layered Oxide Cathodes for Reliable Sodium-Ion Batteries

Toshiya Takuwa^1^, Masataka Yoshimoto^2^, Yuta Kimura^3^, Koji Amezawa^3^, Hitoshi Abe^4,5,6^, Yasuhiro Niwa^4,5^, Satoshi Hiroi^7^, Koji Ohara^7^, Huu Duc Luong^8^, Yasunobu Ando^8^,

Yoshitaka Tateyama^8^, Takashi Nakamura^2^*

**Experimental Section**

**Material synthesis and characterizations**: Na_2/3_Ni_1/3_Mn_2/3_O_2_ was synthesized by solid state reaction method. Na_2_CO_3_ (99%, Kanto Chemical Co., Inc.), NiO (99.9%, Koujundo Chemical Laboratory Co., Ltd.) and Mn_2_O_3_ (99.9%, Koujundo Chemical Laboratory Co., Ltd.) were mixed in a proper ratio with 2mol% excess Na. The mixed powders were sintered at 900^o^C for 15 h in air, and rapidly cooled in an Ar-filled grove box. The morphology of powder samples were characterized by scanning electronic microscopy (TM-4000 Plus III, Hitachi High-Tech Corp.) and energy-dispersive X-ray spectroscopy (AZtecLiveOne Xplore, Oxford Inst.). The crystal structure of pristine and oxygen-deficient samples was characterized by X-ray diffraction (Miniflex, Rigaku, Cu *K*α radiation) in the 2*θ* range of 10 – 80°. Synchrotron XRD was carried out at BL13-XU, SPring-8. Rietveld refinement was performed. The oxygen occupation was assumed as 1 (fully occupied) in pristine materials, and in oxygen-deficient samples, the occupation is assumed as calculated by coulometric titration. X-ray absorption spectroscopy was carried out at BL12-C, Photon Factory and BL-11, Synchrotron Radiation Center, Ritsumeikan University. Ni *L*_III_-edge spectra were recorded in partial fluorescence yielding and total current yielding mode, and Mn *L*_III_-edge was detected in inversed partial fluorescence yielding (IPFY) mode. Transition metal K-edge spectra was recorded by transmission measurement.

**Electrochemical test**: Composite electrodes consisting of 80 wt% active cathode material, 10 wt% acetylene black (AB) conductor and 10 wt% polyvinylidene fluoride (PVDF) binder on Al foil as current collector was assembled to batteries in an argon-filled glove box. The batteries used metallic sodium as anode and 1 mol L^-1^ NaFP_6_-ethylene carbonate/dimethyl carbonate (EC/DEC, 1:1 v/v%) as electrolyte. Galvanostatic charge and discharge cycling was performed in the voltage range of 2.0-4.2 V at 25^o^C.

**Oxygen Coulometric Titration**. The electrochemical technique, coulometric titration, was applied for evaluating oxygen release behavior at 400 to 600 ^o^C. Scheme S1 shows the illustration of the oxygen coulometric titration cell used in this work. The cell was assembled with tubular yttrium stabilized zirconia (YSZ) as an oxide-ion conducting solid-electrolyte. The target sample was placed in the YSZ tube and the Au inner current collector was connected. The tube was filled with Ar and sealed. Porous Au outer electrode was placed on outside of the YSZ tube as a counter electrode. The amount of released oxygen from the sample, *δ*, was calculated from the electric charge, *C*, passed through the cell by

| $\delta+\Delta M_{O(gas)}=\frac{C}{2nF}$ | S1 |
| --- | --- |

where $\Delta M_{O(gas)}$, *n* and *F* are the molar change of oxygen gas in the coulometric cell, the mole of the sample and the Faraday constant, respectively. Compensation of residual O_2_ molecules in the coulometric cell is needed in high *P*(O_2_) range where *P*(O_2_) is larger than 10^-3^ bar. The values of the electromotive force, *E*, at equilibrium state were recorded, in order to obtain the equilibrium relation between *P*(O_2_) and oxygen content. equilibrium *P*(O_2_) of the sample, *P*(O_2_)_sample_, can be obtained by the Nernst equation.

| ${P\left( O_{2} \right)}_{s\mathrm{ample}}={P\left( O_{2} \right)}_{\mathrm{ref}}\exp\left( -\frac{4FE}{RT} \right)$ | S2 |
| --- | --- |

where *R*, *T*, and *P*(O_2_)_ref_ represent the gas constant and the temperature, and *P*(O_2_) at the outer electrode exposed to air (*P*(O_2_) = 0.21 bar), respectively.

1mol% and 5mol% oxygen-deficient samples were prepared by electrochemical oxygen pump with the oxygen coulometric titration cells. After fabricating of the call, necessary electric charge was applied to extract lattice oxygen from the sample at 600 ^o^C, and then, the whole cell was quickly cooled to freeze oxygen content in the sample. Although the cooling rate is not high, oxygen intake from inner atmosphere during cooling is considered to be negligible because *P*(O_2_) in the surrounding atmosphere is low enough.


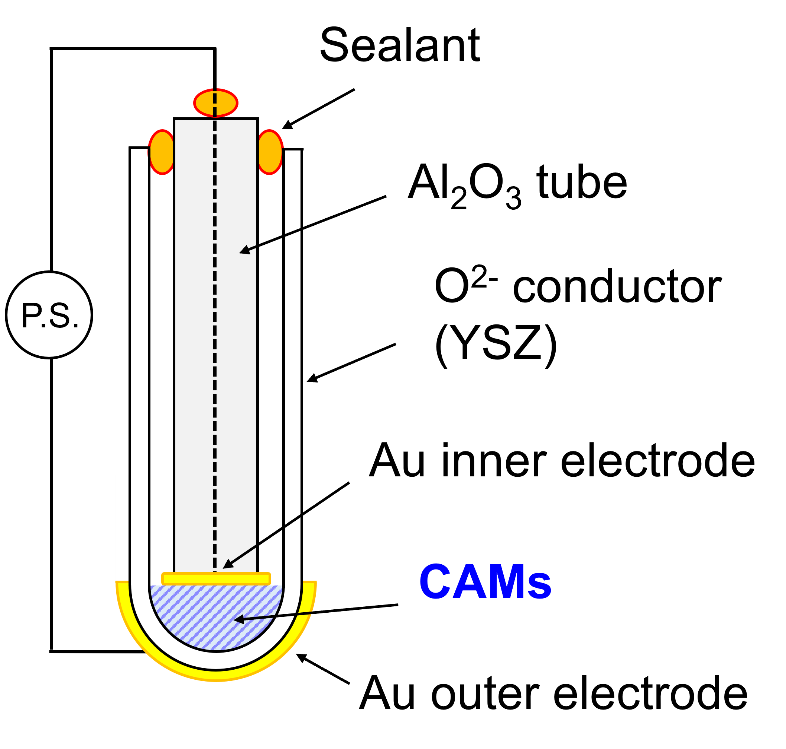


Scheme S1. The illustration of the oxygen coulometric titration cell.

**Calculation section**

All of the density-functional-theory (DFT) based first-principles calculations were implemented by employing the Vienna *Ab initio* Simulation Package (VASP) with the projector augmented wave (PAW) pseudopotentials^[44]^. The Heyd–Scuseria–Ernzerhof exchange-correlation functional (HSE06, 𝛼=1/4) ^[46,49]^ was employed for the layered oxide cathode material^[50,51]^. A cut-off energy of 520 eV and a k-mesh of 1×1×1 were used. The cell relaxation calculations were converged until the stress tensor was minimum and the residual forces were less than 2×10^-2^ eV/Å. In the search of [Na-Ni-Mn] ordering for Na_2/3_Ni_1/3_Mn_2/3_O_2_, $\sqrt{3}$×$\sqrt{3}$ ×1 supercell of P2-NaMnO_2_ was used as the parent structure. 24 unique structures were generated using pymatgen^[52]^ modules, then their cells were fully optimized by the HSE06 method to obtain the lowest energy structure for the next step. The lattice constants, bonds length and magnetic moments of stoichiometric and Na-deficient structure are indicated in Table S1.

**Table S1.** Lattice constants, bond length, and magnetic moments obtained for the stoichiometric and Na-deficient structure obtained by the HSE06 method.

| Structure (HSE06 method) | | Stoichiometric | Na-deficient |
| --- | --- | --- | --- |
| Lattice constant | *a* (Å) | 2.89 | 2.86 |
|  | *b* (Å) | 2.88 | 2.87 |
|  | *c* (Å) | 10.87 | 11.07 |
|  | V (Å^3^) | 78.40 | 78.72 |
| Average bond length (Å) | Ni^2+^O | 2.06 | 2.05 |
|  | Ni^3+^O | -- | 1.96 |
|  | Mn^4+^O | 1.91 | 1.91 |
| Magnetic moment (μ_B_) | Ni^2+^ | 1.71 | 1.70 |
|  | Ni^3+^ | -- | 0.92 |
|  | Mn^4+^ | 3.00 | 3.00 |

For oxygen extraction investigation, the 2$\sqrt{3}$×2$\sqrt{3}$×1 Na-stoichiometric Na_16_Ni_8_Mn_16_O_48_ (case 1) and Na-deficient Na_14_Ni_8_Mn_16_O_48_ (case 2) supercells were ultilized. For each case, 48 different oxygen defect configurations were constructed by removing a single oxygen atom at different oxygen sites, and then fully optimized to search for the supercell of the most stable oxygen defect structure. The projected densities of states (pDOS) of the most stable structures were estimated to examine the valence band maximum (VBM) and conduction band minimum (CBM) states of each element in the material. After the model construction, we calculated the formation enthalpy $\Delta H_{O}$ of a single oxygen vacancy using equation (S3):

| $\Delta H_{O}(\mathrm{eV})=E_{\mathrm{Na}_{n}\mathrm{Ni}_{8}\mathrm{Mn}_{16}O_{47}}+\mu_{O}(P(O_{2}), T)-E_{\mathrm{Na}_{n}\mathrm{Ni}_{8}\mathrm{Mn}_{16}O_{48}}$ | S3 |
| --- | --- |

where, *E_M_* stands for total energy of the structure *M. n*=16 and 14 for case (1) and case (2), respectively. $\mu_{O}(P(O_{2}), T)$ is the chemical potential of gaseous O_2_ molecule, estimated by the following equation^[48]^:

| $\mu_{O}(P(O_{2}), T)=\frac{1}{2}\left[ E_{O_{2}}^{\mathrm{total}}+\tilde{\mu}_{O_{2}}\left( T, P^{0} \right)+k_{B}T\ln\left( \frac{P(O_{2})}{P^{0}} \right) \right]$ | S4 |
| --- | --- |

in which, $\tilde{\mu}_{O_{2}}\left( T, P^{0} \right)$depends on temperature *T* and pressure. Thus, $\tilde{\mu}_{O_{2}}\left( T, P^{0} \right)$is calculated with the experimental JANAF thermochemical tables^[53]^. *P*^0^ is the referenced oxygen pressure (1 atm), *k_B_* is the Boltzmann constant. At $P(O_{2})$ = 0.2 atm, and *T* = 400 K, $\mu_{O}(P(O_{2}), T)$ = 6.97 eV.

**Theoretical relation between defect equilibrium model and thermodynamic parameters**

When we assume itinerant electron and oxygen vacancy as major defect species generated during oxygen release, Gibbs free energy, *G*, of Na_2/3_Ni_1/3_Mn_2/3_O_2_ can be expressed by Gibbs free energy in the standard state, $G^{^{\circ}}$, and pseudo-chemical potential of component *i*, $\mu_{i}$, by

| $G=G^{^{\circ}}+\sum_{i} \chi_{i}\mu_{i}=G^{^{\circ}}+\sum_{i} \chi_{i}\mu_{i}^{^{\circ}}+\sum_{i} RT\chi_{i}\ln\gamma_{i}\chi_{i}$ | S5 |
| --- | --- |

where $\chi_{i}$, $\mu_{i}^{^{\circ}}$ and $\gamma_{i}$ are the molar concentration of *i*, the standard chemical potential of *i*, and the activity coefficient of *i*, respectively. Here, we assume $V_{\mathrm{Na}}^{'}$, $\mathrm{Na}_{\mathrm{Na}}^{\times}$, $\mathrm{Ni}_{\mathrm{TM}}^{'}$ (Ni^2+^), $\mathrm{Mn}_{\mathrm{TM}}^{\cdot}$ (Mn^4+^), $V_{O}^{\cdot\cdot}$, $O_{O}^{\times}$ and $e^{'}$ as defect species and defect concentration of $V_{O}^{\cdot\cdot}$, $O_{O}^{\times}$ and $e^{'}$ depends on δ. Then, oxygen chemical potential, $\mu_{O}$, is obtained by

| $\mu_{O}=\frac{\partial G}{\partial(2-\delta)}=-\frac{\partial G}{\partial\delta}=-\sum_{i} \frac{\partial\chi_{i}}{\partial\delta}\mu_{i}^{^{\circ}}-RT\sum_{i} \frac{\partial\chi_{i}}{\partial\delta}\ln\gamma_{i}-RT\frac{\partial}{\partial\delta}\sum_{i} \chi_{i}\ln\chi_{i}$ | S6 |
| --- | --- |

where $G^{^{\circ}}$, $\mu_{i}^{^{\circ}}$ and $\gamma_{i}$ are considered to be independent on oxygen content. The configurational entropy, $S_{(conf.)}$, of Na_2/3_Ni_1/3_Mn_2/3_O_2_ is expressed

| $S_{(conf.)}=k\left[ \sum_{i} \ln\frac{N_{A}!}{\chi_{\mathrm{Na}_{\mathrm{Na}}^{\times}}N_{A}!\chi_{V_{\mathrm{Na}}^{'}}N_{A}!}+\sum_{i} \ln\frac{N_{A}!}{\chi_{\mathrm{Ni}_{\mathrm{TM}}^{'}}N_{A}!\chi_{\mathrm{Mn}_{\mathrm{TM}}^{\cdot}}N_{A}!}+\sum_{i} \ln\frac{2N_{A}!}{\chi_{O_{O}^{\times}}N_{A}!\chi_{V_{O}^{'}}N_{A}!} \right]$ | S7 |
| --- | --- |

The first, the second and the third terms in eq. S7 represent the combination of the Na site, that of the transition metal site and that of the oxygen site in Na_2/3_Ni_1/3_Mn_2/3_O_2_, respectively. Considering $R=kN_{A}$ and Starling’s formula, partial molar entropy of configurational entropy, $s_{O(conf.)}$, is obtained by

| $s_{O(conf.)}=\frac{\partial S_{(conf.)}}{\partial(2-\delta)}=-\frac{\partial S_{(conf.)}}{\partial\delta}=R\frac{\partial}{\partial\delta}\sum_{i} \chi_{i}\ln\chi_{i}$ | S8 |
| --- | --- |

The eq. S8 is the same with the third term in eq. S6 and therefore, we obtain

| $\mu_{O}=-\sum_{i} \frac{\partial\chi_{i}}{\partial\delta}\mu_{i}^{^{\circ}}-RT\sum_{i} \frac{\partial\chi_{i}}{\partial\delta}\ln\gamma_{i}-T\left( s_{O(conf.)}+R\frac{\partial}{\partial\delta}\chi_{e^{'}}\ln\chi_{e^{'}} \right)$ | S9 |
| --- | --- |

$R\frac{\partial}{\partial\delta}\chi_{e^{'}}\ln\chi_{e^{'}}$ is assumed to be the entropy contribution of itinerant electron. Since $\Delta G_{\mathrm{ox}}^{^{\circ}}$, the Gibbs free energy change of the equilibrium reaction between the sample and O_2_(g), $O_{O}^{\times}\leftrightarrow V_{O}^{\cdot\cdot}+2e^{'}+\frac{1}{2}O_{2}(g)$, can be expressed by

| $\Delta G_{\mathrm{ox}}^{^{\circ}}=\mu_{O_{O}^{\times}}^{^{\circ}}-\mu_{V_{O}^{\cdot\cdot}}^{^{\circ}}-2\mu_{e^{'}}^{^{\circ}}-\frac{1}{2}\mu_{O_{2}(g)}^{^{\circ}}$ | S10 |
| --- | --- |

we obtain the following relationship

| $\mu_{O}-\mu_{O}^{^{\circ}}=\Delta G_{\mathrm{ox}}^{^{\circ}}-RT\ln\frac{\gamma_{V_{O}^{\cdot\cdot}}\gamma_{e^{'}}^{2}}{\gamma_{O_{O}^{\times}}}-T\left( s_{(conf.)}^{O}-R\chi_{e^{'}}\ln\chi_{e^{'}} \right)$ | S11 |
| --- | --- |
| $h_{O}-h_{O}^{^{\circ}}=\Delta H_{\mathrm{ox}}^{^{\circ}}-RT\ln\frac{\gamma_{V_{O}^{\cdot\cdot}}\gamma_{e^{'}}^{2}}{\gamma_{O_{O}^{\times}}}$ | S12 |

where $\mu_{O_{2}(g)}^{^{\circ}}=2\mu_{O}^{^{\circ}}$. When the activity coefficients of defect species are considered as unity, meaning that defect interaction is negligibly small, one can obtain, $h_{O}-h_{O}^{^{\circ}}=\Delta H_{\mathrm{ox}}^{^{\circ}}$. As shown in the Table 2 in the main text, $h_{O}-h_{O}^{^{\circ}}=\Delta H_{\mathrm{ox}}^{^{\circ}}$ is achieved in Na_2/3_Ni_1/3_Mn_2/3_O_2_.

**Other Figures and Tables**

**Table S2.** Refined crystallographic parameters by Rietveld analysis for (a) the pristine and (b) the 1 mol% oxygen-deficient Na_2/3_Ni_1/3_Mn_2/3_O_2_.

(a) Pristine Na_2/3_Ni_1/3_Mn_2/3_O_2_. *a* = 2.8721 Å, *c* = 11.079 Å, *α* = 90 ^o^, *β* = 90 ^o^ and *γ* = 120 ^o^. *R*_wp_ =14.1%, *R*_e_ = 2.29%, *S* = 6.15. Values in parentheses were fixed.

| Atom | site | *x* | *y* | *z* | *g* | *B* |
| --- | --- | --- | --- | --- | --- | --- |
| Na | 2c | 1/3 | 2/3 | 1/4 | 0.3826 | 5.201 |
| Na | 2b | 0 | 0 | 1/4 | 0.2840 | 2.031 |
| Mn | 2a | 0 | 0 | 0 | (0.67) | 0.3727 |
| Ni | 2a | 0 | 0 | 0 | (0.33) | 0.3727 |
| O | 4f | 1/3 | 2/3 | 0.5895 | (1.0) | 0.5244 |

(b) 1 mol% oxygen-deficient Na_2/3_Ni_1/3_Mn_2/3_O_2_. *a* = 2.9303 Å, *c* = 11.327 Å, *α* = 90 ^o^, *β* = 90 ^o^ and *γ* = 120 ^o^. *R*_wp_ = 10.4%, *R*_e_ = 2.44%, *S* = 4.25. Values in parentheses were fixed.

| Atom | site | *x* | *y* | *z* | *g* | *B* |
| --- | --- | --- | --- | --- | --- | --- |
| Na | 2c | 1/3 | 2/3 | 1/4 | 0.4225 | 5.525 |
| Na | 2b | 0 | 0 | 1/4 | 0.2442 | 3.251 |
| Mn | 2a | 0 | 0 | 0 | (0.67) | 0.4273 |
| Ni | 2a | 0 | 0 | 0 | (0.33) | 0.4273 |
| O | 4f | 1/3 | 2/3 | 0.5882 | (0.99) | 0.7246 |


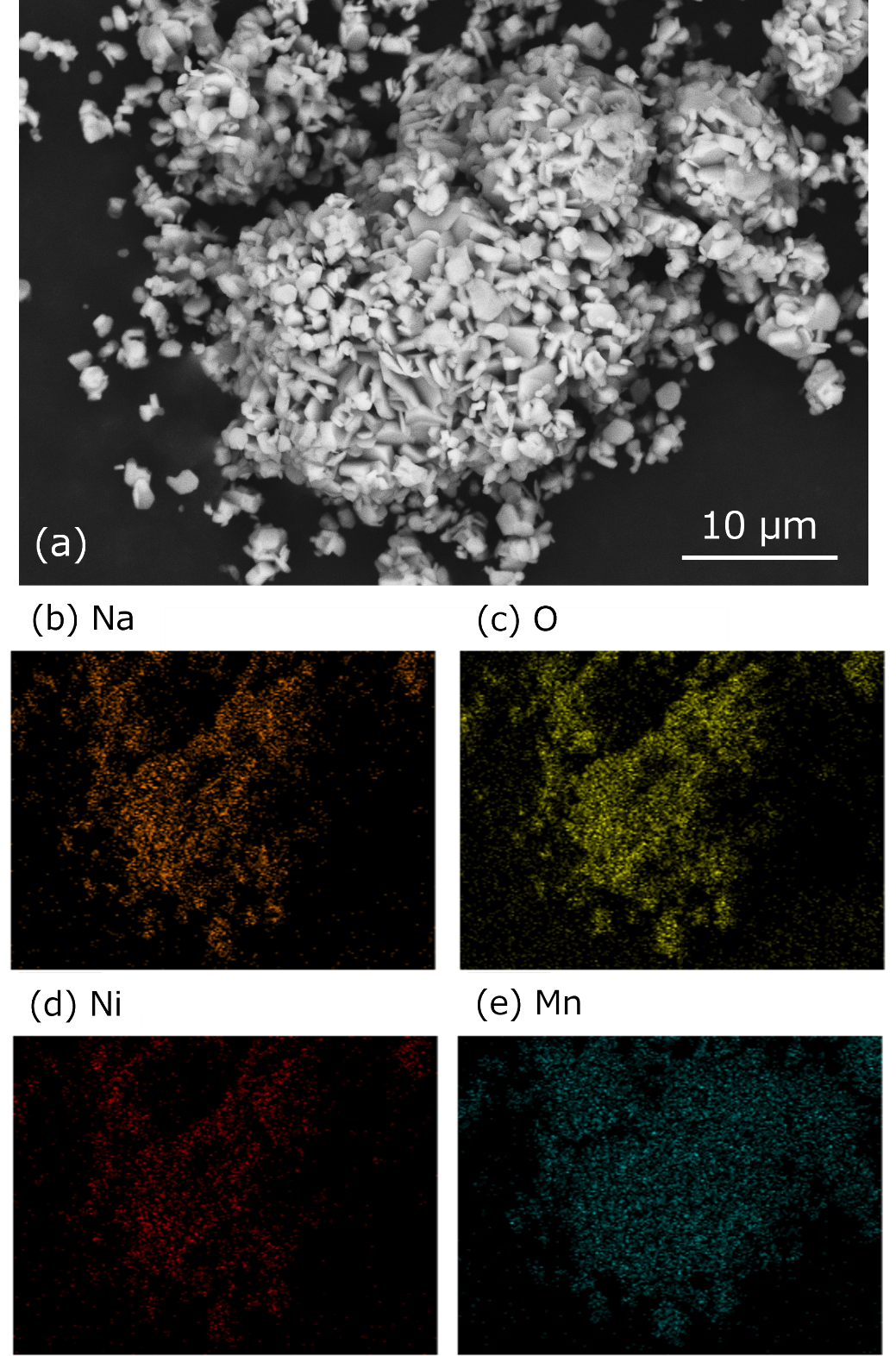


**Figure S1.** (a) SEM image of Na_2/3_Ni_1/3_Mn_2/3_O_2_ particles and EDX mappings of (b) Na, (c) O, (d) Ni and (e) Mn.


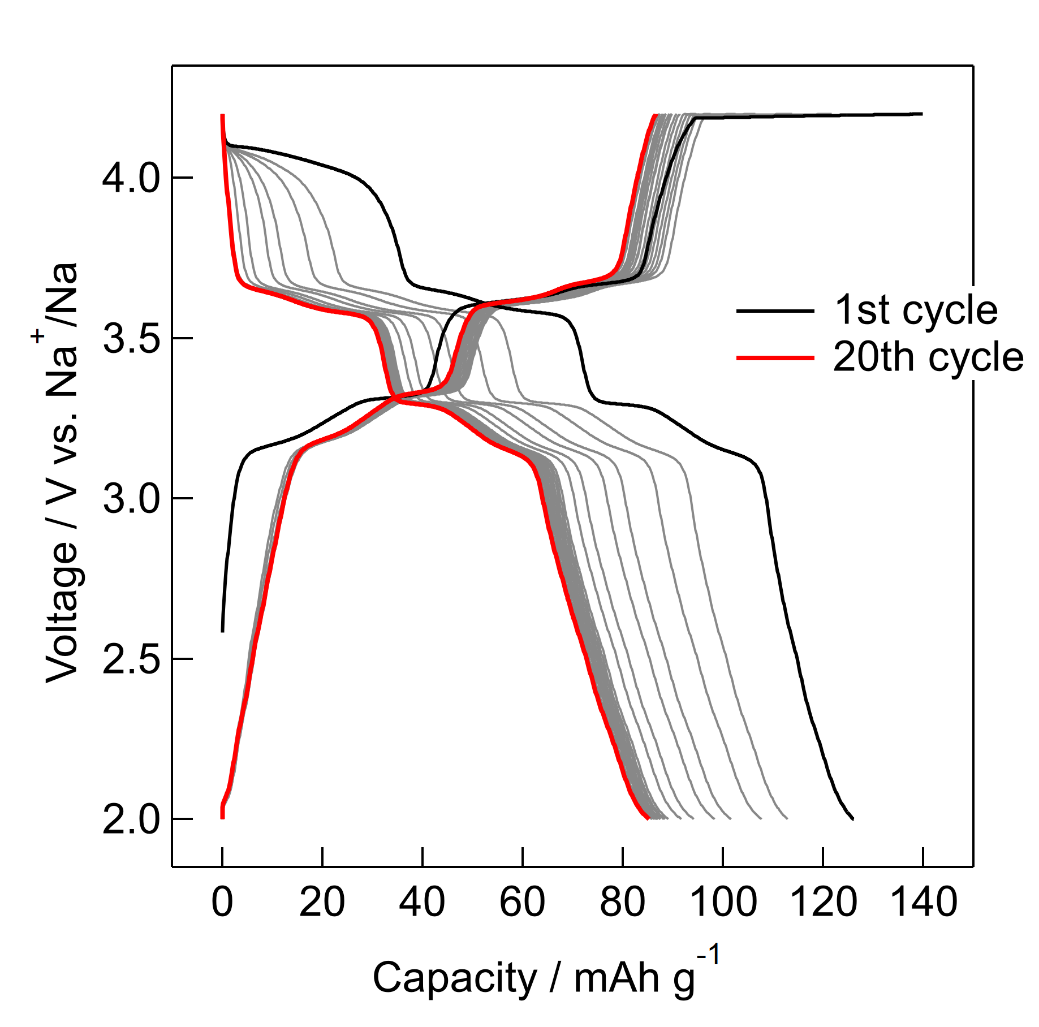


**Figure S2.** Charge and discharge curves of Na_2/3_Ni_1/3_Mn_2/3_O_2_ between 2.0 - 4.2 V vs. Na^+^/Na. The battery test cell was composed of the composite cathode / 1 molL^-1^ NaPF_6_ EC-DEC / Na metal.


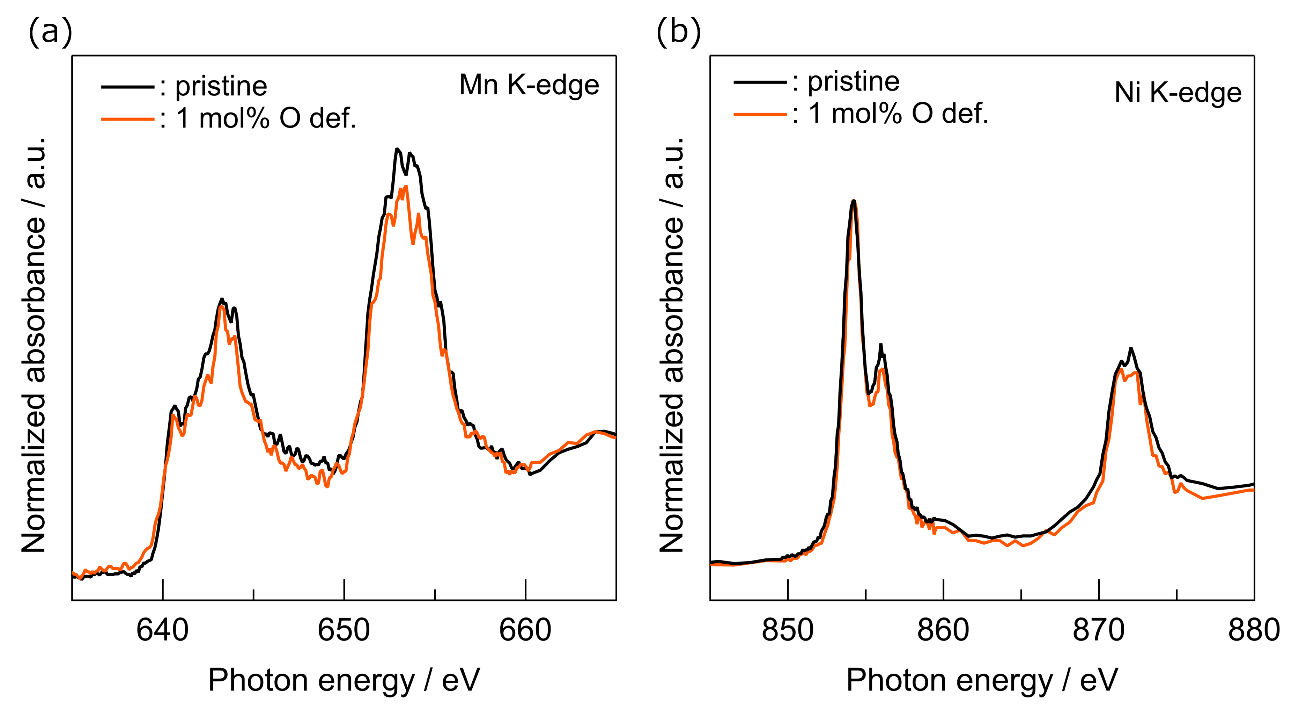


**Figure S3**. X-ray absorption spectra at (a) Mn L-edge and (b) Ni L-edge of the pristine and the 1mol% oxygen-deficient Na_2/3_Ni_1/3_Mn_2/3_O_2_.


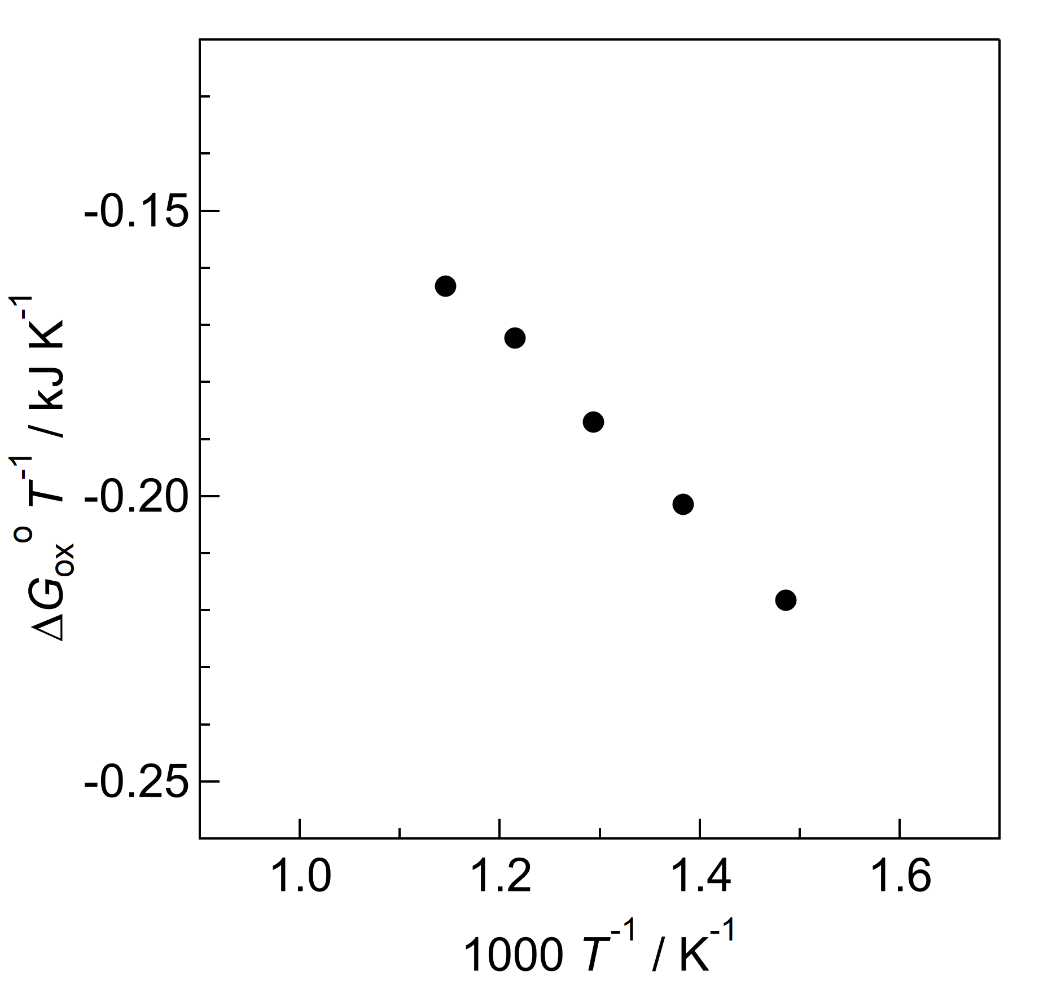


**Figure S4**. Gibbs-Helmholtz plot of $\Delta G_{\mathrm{ox}}^{^{\circ}}$ for Na_2/3_Ni_1/3_Mn_2/3_O_2_. The obtained enthalpy change, $\Delta H_{\mathrm{ox}}^{^{\circ}}$, is about 1.7 eV, agreeing with $h_{O}-h_{O}^{^{\circ}}$ obtained by thermodynamic analysis.


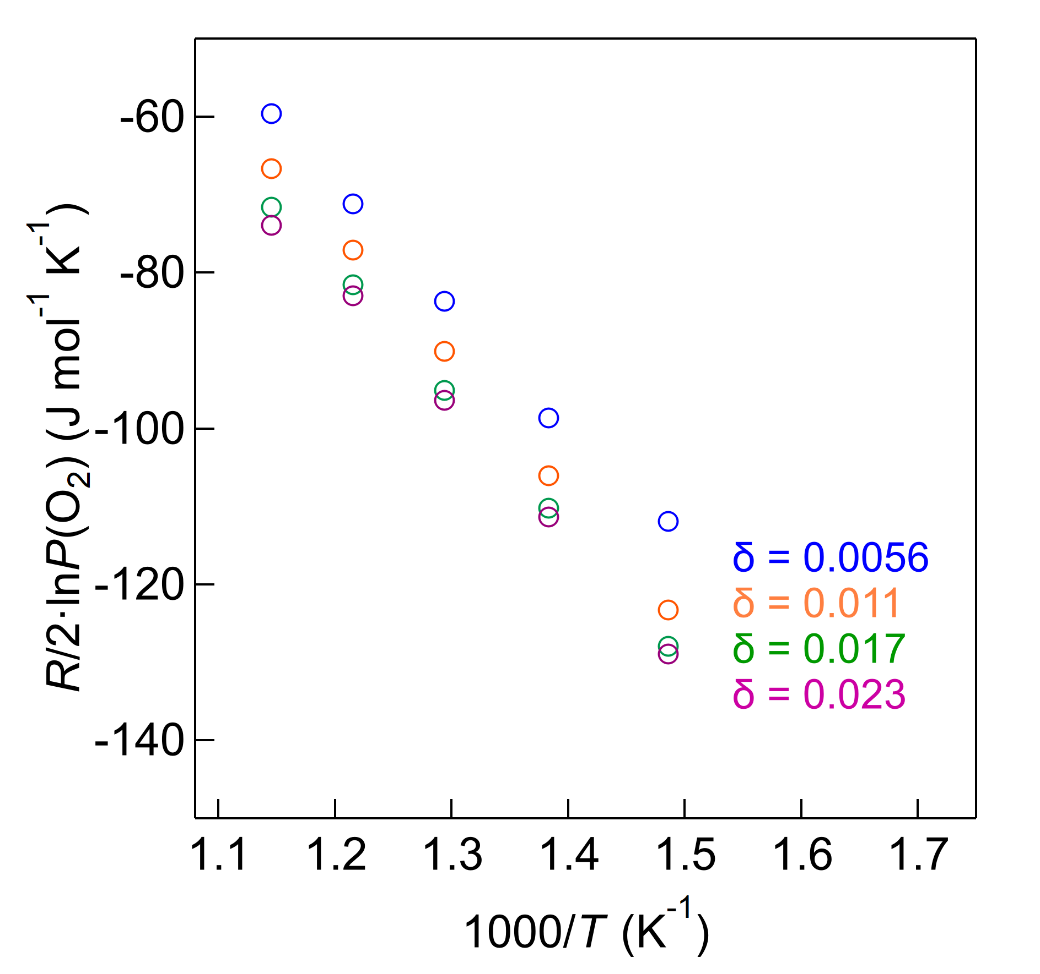


**Figure S5**. Gibbs-Helmholtz plot from *T*-δ-*P*(O_2_) relation of Na_2/3_Ni_1/3_Mn_2/3_O_2-δ_.

References

(1) Kresse, G.; Hafner, J. *Ab Initio* Molecular Dynamics for Liquid Metals. *Phys. Rev. B* **1993**, *47* (1), 558–561. https://doi.org/10.1103/PhysRevB.47.558.

(2) Heyd, J.; Scuseria, G. E.; Ernzerhof, M. Hybrid Functionals Based on a Screened Coulomb Potential. *J. Chem. Phys.* **2003**, *118* (18), 8207–8215. https://doi.org/10.1063/1.1564060.

(3) Krukau, A. V.; Vydrov, O. A.; Izmaylov, A. F.; Scuseria, G. E. Influence of the Exchange Screening Parameter on the Performance of Screened Hybrid Functionals. *J. Chem. Phys.* **2006**, *125* (22). https://doi.org/10.1063/1.2404663.

(4) Luong, H. D.; Xu, C.; Jalem, R.; Tateyama, Y. Evaluation of Battery Positive-Electrode Performance with Simultaneous Ab-Initio Calculations of Both Electronic and Ionic Conductivities. *J. Power Sources* **2023**, *569*, 232969. https://doi.org/10.1016/j.jpowsour.2023.232969.

(5) Luong, H. D.; Dinh, V. A.; Momida, H.; Oguchi, T. Insight into the Diffusion Mechanism of Sodium Ion–Polaron Complexes in Orthorhombic P2 Layered Cathode Oxide Na _x_ MnO _2_. *Physical Chemistry Chemical Physics* **2020**, *22* (32), 18219–18228. https://doi.org/10.1039/D0CP03208E.

(6) Ong, S. P.; Richards, W. D.; Jain, A.; Hautier, G.; Kocher, M.; Cholia, S.; Gunter, D.; Chevrier, V. L.; Persson, K. A.; Ceder, G. Python Materials Genomics (Pymatgen): A Robust, Open-Source Python Library for Materials Analysis. *Comput. Mater. Sci.* **2013**, *68*, 314–319. https://doi.org/10.1016/J.COMMATSCI.2012.10.028.

(7) Reuter, K.; Scheffler, M. Composition and Structure of the of the RuO2⁢(110) Surface in an O2 and CO Environment: Implications for the Catalytic Formation of CO2. *Phys. Rev. B* **2003**, *68* (4), 45407. https://doi.org/10.1103/PhysRevB.68.045407.

(8) Chase, M. W. NIST‐JANAF Thermochemical Tables for Oxygen Fluorides. *J. Phys. Chem. Ref. Data* **1996**, *25* (2), 551–603. https://doi.org/10.1063/1.555992.
